# Intensive tobacco cultivations, a possible public health risk?

**DOI:** 10.1007/s11356-019-04239-6

**Published:** 2019-03-19

**Authors:** Giuseppe Michele Masanotti, Elia Abbafati, Elena Petrella, Simone Vinciguerra, Fabrizio Stracci

**Affiliations:** Department of Experimental Medicine, University of Perugia, pz. Lucio Severi 1, 06129 Perugia, Italy

**Keywords:** Tobacco, Cultivations, Nicotine, Public health, Environment, Sustainability

## Abstract

The cultivation of tobacco has serious consequences for the environment: it impoverishes the soil by assimilating its nutrients, it involves an intensive use of highly polluting pesticides, it perturbs the ecosystem through deforestation, and it releases nicotine into the environment, which is toxic for humans. Italy is the first producer of raw tobacco in Europe and the Valtiberina area is among the most profitable. The first cultivations can be reconducted to the period around 1400. The objective of this experimental work is to verify the sustainability of tobacco cultivation near other crops using nicotine as an indicator. The nicotine on medicinal and wild plants adjacent to tobacco crops has been analyzed, assessing whether it is present or not and which is the concentration. To measure the nicotine present with ultra-high-performance liquid chromatography (UHPLC), LC/MS (liquid chromatography-mass spectrometry) method was used with LOQ (quantification limit) of 0.005 mg/kg. A total of 300 lots of aromatic herbs were sampled, and nicotine was detected in 82.3% of the samples in 2015 and 62.9% in 2016. Furthermore, in 2015, 121 samples of wild material were analyzed, of which 88.4% showed traces of nicotine. These first results indicate a possible potential threat for the population health. This shows that the tobacco cultivation should not be in close proximity to other plantation destined for nutrition, neither for man and nor animals. The elevated impact of nicotine on the ecosystem has negative consequences not only for the economy but it is also a potential public health threat.

## Introduction

The tobacco epidemic is one of the biggest public health threats the world is facing, killing more than seven million people a year. More than six million of those deaths are the result of direct tobacco use, while around 890,000 are the result of non-smokers being exposed to second-hand smoke (WHO 2017). According to Mathers, without a concrete commitment, the total deaths related to tobacco are expected to grow to more than eight million a year by 2030, equivalent to 10% of annual deaths worldwide (Mathers and Loncar 2006). Tobacco use is a threat to every person, regardless of sex, age, race, cultural, or educational background; it weighs heavily on national economies through increasing health costs and decreasing productivity. About 80% of premature deaths related to tobacco occur mainly in several low- and middle-income countries (WHO 2017).

Tobacco cultivation has serious environmental consequences, such as loss of biodiversity, erosion and soil degradation, water pollution, and a significant increase of carbon dioxide in the atmosphere (Lecours et al. 2012).

Tobacco production involves the use of chemicals such as pesticides, fertilizers, and growth regulators that can pollute sources of drinking water and groundwater; it also impoverishes the soil by assimilating more nitrogen, phosphorus, and potassium than other cultures. The soil will collapse in a few years with this massive and constant pollution, so it is necessary to move crops elsewhere or to rejuvenate the soil by rotating the crops (Lecours 2014).

Although the literature available on the subject is poor, it has shown that tobacco plantations cause environmental degradation and disruption of the ecosystem not only because of the intensive use of pesticides but also because of deforestation needed to increase the availability of fertile land (Lecours et al. 2012).

Every year in the world, 4.3 million hectares of land are used for tobacco plantations, resulting in a global deforestation between 2% and 4%. Moreover, the entire tobacco industry (cultivation, processing, and distribution) produces more than 2 million tones of solid waste. It is estimated that from 1995 to 2015, global tobacco production will deposit a total of 45 million tonnes of solid waste, 6 million tons of nicotine waste, and over 4 million tons of chemical waste (Novotny and Zhao 1999).

Italy is Europe’s first producer of raw tobacco, with a 27% share and total volumes of around 50,000 tones; 97% of tobacco is cultivated in only four regions: Campania, Umbria, Veneto, and Tuscany; but until the eighties of the last century, also the province of Lecce, in Apuglia, strongly contributed to the development of tobacco farming (Montinari et al. 2018). With the exception of Eastern tobaccos, all tobacco varieties are grown in Italy; these crops create employment for more than 50,000 workers (Italian Ministry of Agriculture 2018).

The Upper Tiber Valley is one of the areas where the cultivation of tobacco is more substantial and where approximately 109,000 people live. The massive or intensive use of chemicals for the cultivation of tobacco in this area is on the local agenda, which brought to the introduction of standard procedures that led to the current tolerance thresholds for health risk in the local community. In terms of exposure risk, all the citizens that live in the valley have the same level of risk of the tobacco growers, due to the geographical and geological conformation of the Upper Tiber Valley (Alunni 2017).

During cutting, manual harvesting, and manual loading, up to 89% of workers can contract the “green tobacco sickness” (GTS), caused by a nicotine absorption into the skin and the respiratory system. The nicotine is a contact poison that gathers on the surface of leaves to protect the plant from insects especially when it is wet with rain or dew. Therefore, in the early hours of the morning, or after rain, there is an increased risk of contracting this sickness, which does not cause an increase in mortality or long-term morbidity, but it causes an important discomfort and a reduction in the productivity of workers.

Symptoms of intoxication include drooling, nausea, vomiting, migraine, diarrhea, respiratory distress, heart rate changes, and increased blood pressure, and it is for these reasons, and not just for smoking damage, that tobacco is cataloged in the poison of toxic plants for humans. (Arcury et al. 2003a, b) In a recent review, conducted on GTS, the prevalence of GTS varies from 8.2 to 47% globally (Fotedar and Fotedar 2017).

Nicotine, also for this reason, is considered to be dangerous by the European Directive 2008/1272/CE (essentially referring to commercial products containing nicotine); based on this directive, if nicotine reaches a watercourse, drainage system, soil, or vegetation, it is strongly suggested to notify the competent authorities (Regional Environment Agency, Local Health Agency, Forrestal Police).

They are not just smokers who assimilate tobacco substances; since nicotine is passed on the soil, on medicinal plants, and aromatic and perfumed herbs, all the popultion is at risk.

Medicinal plants are a natural resource that has always been used and have been subject to a renewed and growing cultural and economic interest due to their properties, which enable them to be used in various fields, including herbal, pharmaceutical, and cosmetics. (WHO 1999, 2003, 2007, 2009)

According to WHO estimates, these plants constitute a therapeutic help for about 80% of the world’s population and provide the active principles and adjutants used in about 25% of existing drugs (Ekor 2014).

In this study, it was investigated if the concentration of nicotine is present on wild plants spontaneously grown in the soil and the concentration of nicotine on medicinal plants both grown near the tobacco crops in the area of the Valtiberina. It was assessed whether the regulatory limits were respected, with the aim to verify the environmental sustainability of the tobacco cultivations.

## Materials and methods

The plant matrices collected in Tuscany and Umbria, in particular from the territories of Sansepolcro, Anghiari, Citerna, and Città di Castello, have been analyzed in 2015/2016. The aim was to investigate whether nicotine was present and verify, at the same time, if the involvement of tobacco crops and their processing may have affected the data obtained. There are no filtering devices to prevent environmental dispersion of nicotine, which could be present in the air that escapes from the tobacco drying facilities; there are no specific monitoring plans implemented by the competent authorities to assess the presence of alkaloids in fumes and/or vapors dispersed in the environment.

The medicinal plants analyzed during the project in the areas adjacent to tobacco crops and in which parts nicotine is present are the following: hawthorn, leaves (*Crataegus monogyna* L.); peppermint, leaves (*Mentha x Piperita*); Italian helicide, airline parts (*Helichrysum italicum* Roth); chamomile (*Matricaria chamomilla* L.); nettle, leaves (*Urtica dioica* L.); grindelia, airline parts (*Grindelia robusta* Nutt.); plate, leaves (*Plantago lanceolata* L.); passiflora, leaves (*Passiflora incarnata* L.); malva selvatica, airline parts (*Malva sylvestris* L.); Californian escolzia (*Eschscholzia californica* Cham); blackcurrant (*Ribes nigrum* L.); melissa, airline parts (*Melissa officinalis* L.); common altea, root (*Althaea officinalis* L.); buckwheat, flowering plans (*Fagopyrum esculentum* L.); coriander, seeds (*Coriandrum sativum* L.); dandelion, root (*Taraxacum officinale*).

The analytical data obtained were generated using the LC/MS method (liquid chromatography-mass spectrometry) with a quantification limit (LOQ) of 0.005 mg/kg.

### Sampling method

The test sample is extracted with a mixture of basic acetonitrile and centrifuged and transferred into a vial. Nicotine is quantified with an ultra-high-performance liquid chromatography (UHPLC) with reverse phase column and triple quadrupole mass spectrometry detector. The method involves, at first, the homogenization of the sample by grinding. Next, 0.5 g of dried herb sample is weighted and put into the centrifuge tube and 10 g of water is added. Then, 10 mL of acetonitrile basified with NH3 at pH 9 is added and stirred for 1 min. Afterwards, 6 g of anhydrous magnesium sulfate is added, and the whole is agitated for 2 min to avoid the formation of lumps; it is centrifuged for 5 min and the centrifuge tube is placed in dry ice for 30 min; the tube is removed from the dry ice and left to return to room temperature, then it is centrifuged again for 1 min. Subsequently, 6 mL of supernatant is transferred to the PSA (primary and secondary amine) purification tube (150 mg PSA, 900 mg MgSO4); it is shaken for 1 min and centrifuged for another 5 min. Five hundred microliters of the purified product are taken and the internal standard is added, placing them in a self-refillable vial; the whole is injected into UPLC/MSMS and qualitative and quantitative determination is made using the internal standard method (using Nicotine-D4).

The result obtained is multiplied by the dilution; the quantification limit is 0.005 mg/kg, and the range of measurement is between 0.005 mg/kg and 0.10 mg/kg. It was decided to use Hortwiz’s simplified approach with Thompson’s correction in order to estimate uncertain measurement.

## Results

**Fig. 1:**
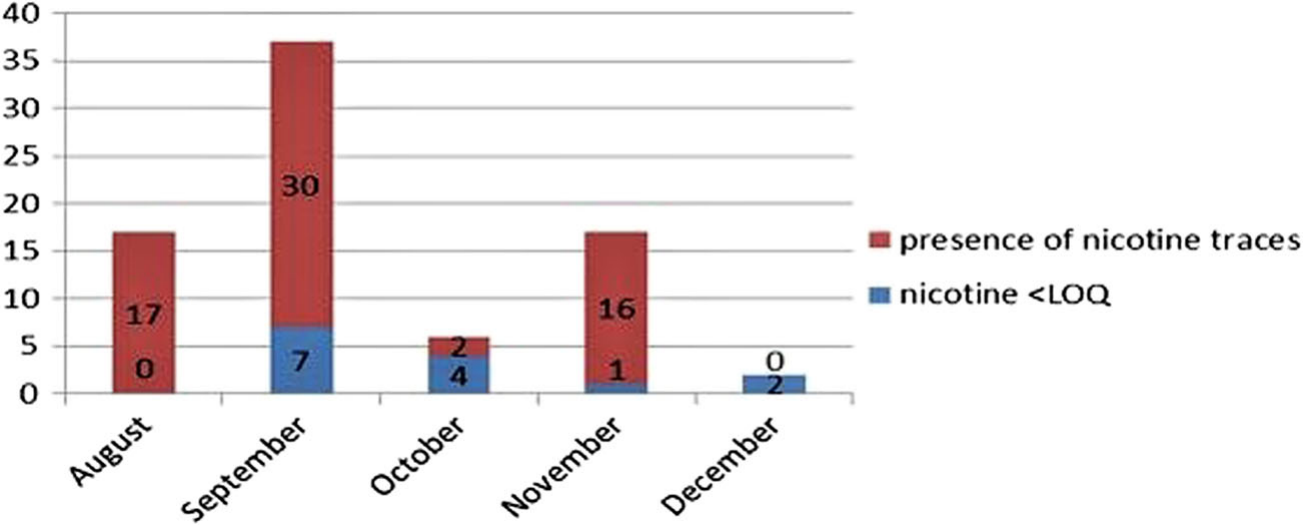
Number of samples of organic aromatic herbs examined from August to December 2015

**Fig. 2:**
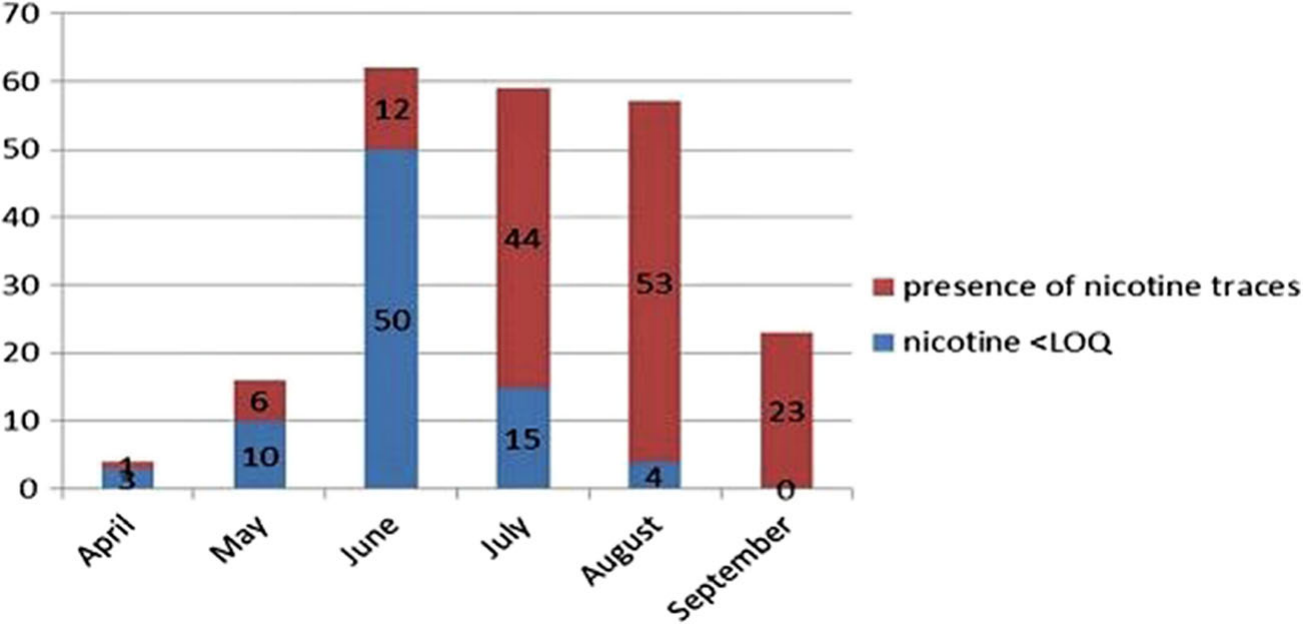
Number of samples of organic aromatic herbs examined from April to September 2016

Figures 1 and 2 show the analytical results obtained by the sampling of organic aromatic herbs in the Valtiberina Umbria and Tuscany regions during the period from 2015 to 2016. Seventy-nine and 221 batches of organic aromatic herbs were examined in 2015 and 2016, respectively. The alkaloid was found in 65 samples (82.3%) in 2015 and in 139 (62.9%) in 2016; of these, 23 lots (29.1%) in 2015 and 28 (12.7%) in 2016 were declared non-compliant because the concentration of nicotine was higher than 0.4 mg/kg. The batches with values below the limit of nicotine are highlighted in blue, whereas those in which nicotine or traces of it has been found are shown in red. The higher concentration of nicotine was observed in the months of August and September when tobacco cultivation was close to ripening and thus at the beginning of the drying processes. That according to the national guidelines must be carried out with a “flue cured” air flow, inside special “bulk-curing” dryers, by hot air forced ventilation (Riquinho and Hennington 2012).

**Fig. 3:**
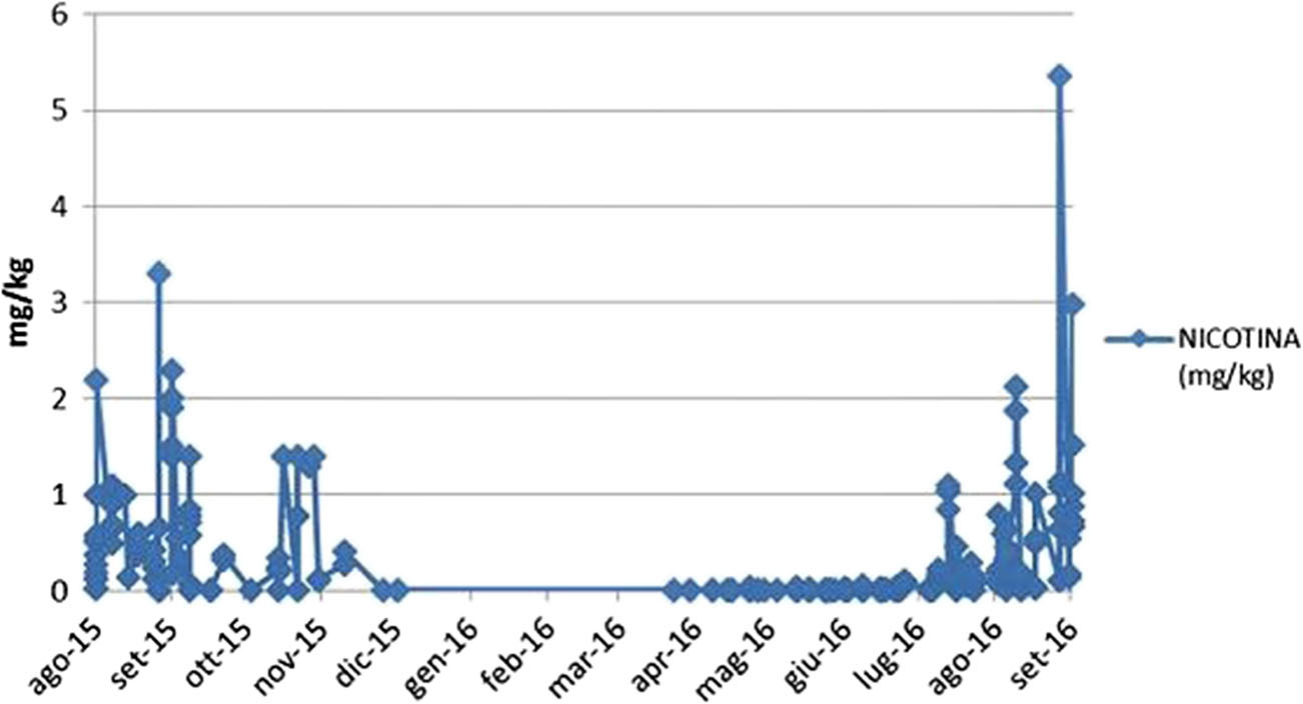
Quantities of nicotine in mg/kg found in organic herbs

Figure 3 shows that in both years, the amount of nicotine was strongly linked to the tobacco production and drying cycle, with an exponential increase in August and September reaching peaks higher than 3 mg/kg in 2015 and 5 mg/kg in 2016.

Both the number of positive lots, in which nicotine is present but falls within the limits allowed by law, and the non-conforming lots, in which nicotine is found in a higher percentage than the limits allowed, are highlighted in Table 1 (Commission Regulations 2011, 2013, 2015)

**Table 1:** Total lots of organic aromatic herbs, 2015/16

	The year 2015 - by 20/08/2015	%	The year 2016 - until 30/09/2016	%
Analyzed lots	79	–	221	–
Positive lots	65	82.3	139	62.9
Positive lots of which it does not conform	23	29.1	28	12.7

**Fig. 4:**
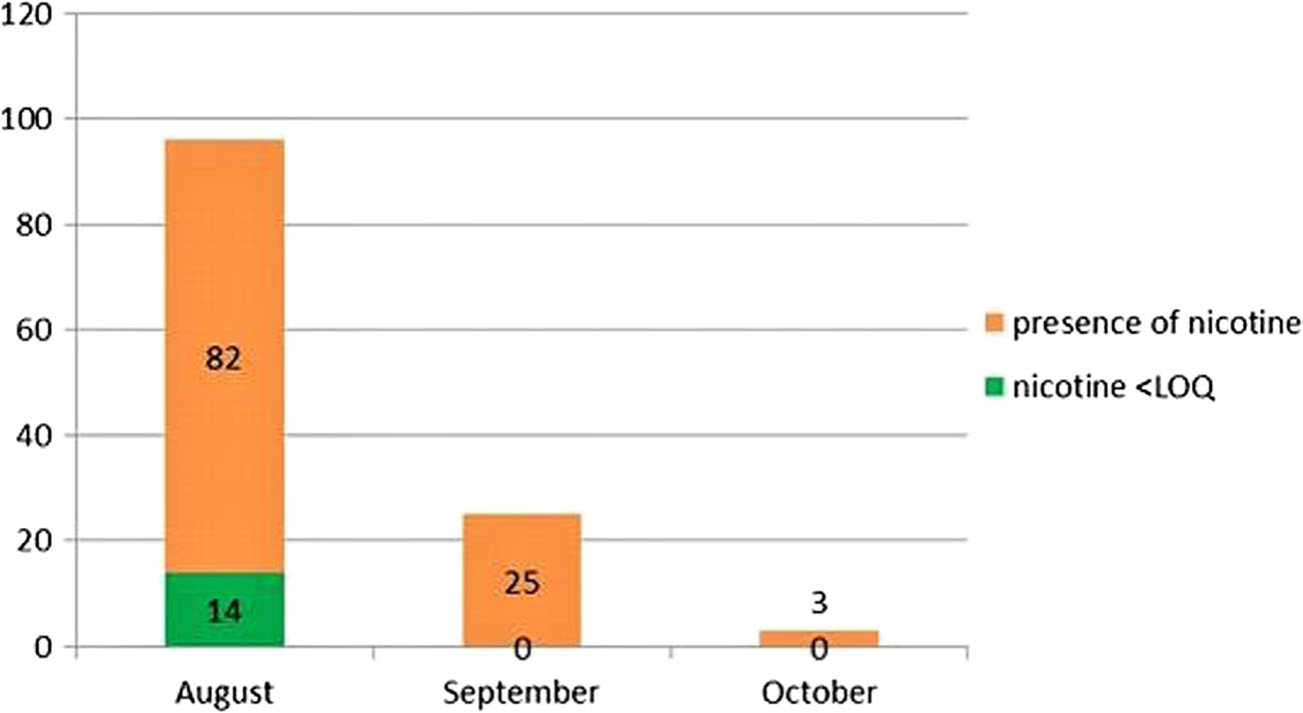
Wild harvest material during 2015

**Fig. 5:**
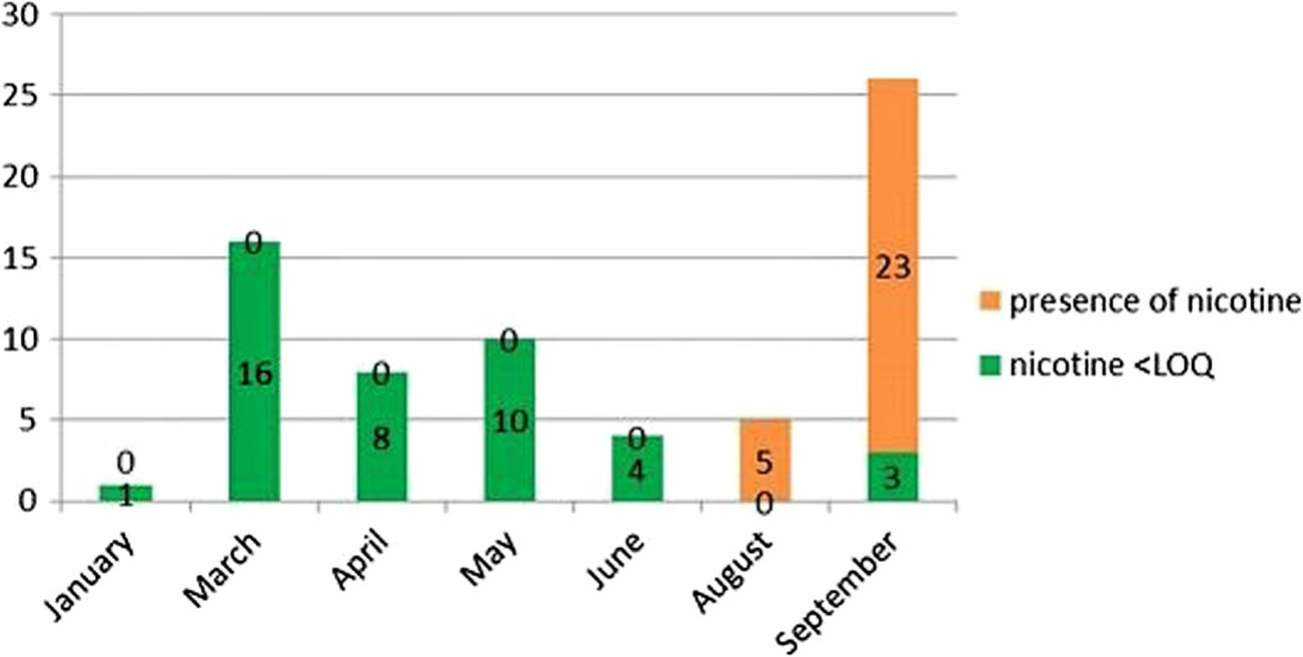
Wild harvest material during 2016

The research is integrated by sampling fresh wild harvested crops in neighboring areas for tobacco crops (Figs. 4 and 5). In 2015, 121 samples were analyzed, of which 107 (88.4%) contained nicotine and 57 of those positive samples (47.1%) had a higher concentration than 0.4 mg/kg; for 2016, the positive samples were 28 (90.3%) on 31 analyzed and 4 (12.9%) were found beyond the threshold value.

The following table shows a significant concentration of nicotine in the plant matrices of wild material harvested in August and September (it coincides with the maximum vegetative growth of tobacco cultivation). During this time of the year, alkaloid was observed in 88% of the samples analyzed in 2015 and in 90% of those analyzed in 2016.

Forty-seven percent of the samples in 2015 showed a presence of nicotine greater than 0.4 mg/kg, the limit value of nicotine concentration allowed for fresh culinary herbs (chives, parsley, celery, basil, etc.) (Table 2).

**Table 2:** Wild harvest material

	August–September 2015	%	August–September 2016	%
Analyzed lots	121	–	31	–
Positive lots	107	88,4	28	90,3
Lots > 0.4 mg/kg	57	47.1	4	12.9

## Conclusions

In this study, the endogenous presence of nicotine as a secondary metabolite was detected in the various cultures examined, classified as medicinal plants and wild plants. Considering the bio-accumulation of nicotine in these crops, it can be inferred that nicotine is present in the ecosystem. However, the phenomenon that leads to the contamination of the different environmental matrices remains, still, unidentified.

The analytical evidence proposed and documented in the “Use of temporary MRLs (Maximum Residue Levels), herbal infusions, spices, canine rose and fresh herbs”, written in 2011 by the European Food Safety Authority (published in the EFSA 2011 9-3 journal), establishes that a natural presence of nicotine in areas where tobacco cultivation is grown extensively is going to take over most of the agricultural land as occurred in the area of Valtiberina Umbra and Tuscany. In fact, investigations have shown that the tendency of nicotine environmental concentration is linked to the tobacco production cycle. Data confirms what EFSA has already indicated: “... investigations have revealed that the presence of nicotine is not limited to Solanaceae and mushrooms, but to a wider variety of crops where it was not expected to be found. The reasons for the presence of nicotine in the cultures under consideration [...] may be the result of tobacco cultivation in nearby fields”.

In regard to the high environmental impact of tobacco cultivation for the use of significant quantities of chemical products (soil disinfectants, fertilizers, etc..), the adoption of the best environmental friendly farming practices should be advised. A more efficient use of natural resources can be achieved through sustainability, only with an important reduction in pesticides. Having established the unsustainability of tobacco plantations near homes, other crops, or farms, guidelines and regulations should be issued in order to protect citizens who live or work in these areas. As of now, there is no legislation on threshold distances from tobacco crops; therefore, the population is exposed to the harmful effects that nicotine causes.

According to EU Regulations n. 812/2011 and EU n. 1004/2013 and the subsequent EU n. 401/2015, the maximum limits currently in place represent a significant precautionary action for the protection of consumers. In this particular case, a significant number of matches, namely, 23 lots in 2015 and 28 lots in 2016, were destroyed following the limits set by the regulation. In the absence of regulatory constraints, the only possibility of intervention is represented by the ASL (Local Health Agencies) or the ARPA (Regional Environmental Agency) monitoring and controlling actions. The complaints about the high pollution cannot fail to make who is called to administer the territory think about a possible solution. The interest of the inhabitants of the Upper Tiber area on this issue shows how the level of awareness has increased. The idea of a diversification of crops has begun to reach farmers.

A serious regional planning may, therefore, begin a local conversion process (Campania Region 2015), which, follows the recent EU community policies, must be one of the priority objectives of the political forces and economic world.

In Italy, as in many other countries, the initiatives are multiplying. The UN Agenda 2030, signed by 193 countries on 25 September 2015, establishes 17 ambitious objectives of sustainable development, calling all of us to create opportunities to improve well-being for all, respecting the environment from which we draw our lives (United Nations 2015).
